# Death from Chloroform

**Published:** 1890-01

**Authors:** 


### Death From Chloroform.

A death from the inhalation of chloroform occurred at the Richmond Hospital, London, Oct. 12. The patient was a woman, who was about to undergo amputation of the thumb. She had taken a very few inspirations when her face was observed to become deeply congested. The administration of the anæsthetic was at once stopped, artificial respiration was set up, and the external jugular vein was opened, but she never rallied. At the autopsy examination the heart was found to be infiltrated with fat and the brain to be congested. The coroner's jury found that the anæsthetic was skillfully administered.—*British Medical Journal*, Oct. 12, 1889.

